# Extracellular DNA release confers heterogeneity in *Candida albicans* biofilm formation

**DOI:** 10.1186/s12866-014-0303-6

**Published:** 2014-12-05

**Authors:** Ranjith Rajendran, Leighann Sherry, David F Lappin, Chris J Nile, Karen Smith, Craig Williams, Carol A Munro, Gordon Ramage

**Affiliations:** Infection and Immunity Research Group, Glasgow Dental School, School of Medicine, College of Medical, Veterinary and Life Sciences, University of Glasgow, 378 Sauchiehall Street, Glasgow, G2 3JZ UK; Institute of Healthcare Associated Infection, School of Health, Nursing and Midwifery, University of the West of Scotland, Paisley, UK; Aberdeen Fungal Group, Institute of Medical Sciences, Foresterhill, University of Aberdeen, Aberdeen, AB25 2ZD UK

**Keywords:** *Candida albicans*, Biofilm, eDNA, Chitinases

## Abstract

**Background:**

Biofilm formation by *Candida albicans* has shown to be highly variable and is directly associated with pathogenicity and poor clinical outcomes in patients at risk. The aim of this study was to test the hypotheses that the extracellular DNA release by *C. albicans* is strain dependent and is associated with biofilm heterogeneity.

**Results:**

Initially, biofilm formed by *C. albicans* high biofilm formers (HBF) or low biofilm formers (LBF) were treated with DNase to find whether eDNA play a role in their biofilm formation. Digestion of biofilm eDNA significantly reduced the HBF biofilm biomass by five fold compared to untreated controls. In addition, quantification of eDNA over the period of biofilm formation by SYBR green assay demonstrate a significantly higher level of 2 to 6 fold in HBF compared to LBF. Biochemical and transcriptional analyses showed that chitinase activity and mRNA levels of chitinase genes, a marker of autolysis, were upregulated in 24 h biofilm formation by HBF compared to LBF, indicating autolysis pathway possibly involved in causing variation. The biofilm biomass and eDNA release by single *(∆cht2, ∆cht3*) and double knockout (*∆cht2/∆cht3)* chitinase mutants were significantly less compared to their parental strain CA14, confirming the role of chitinases in eDNA release and biofilm formation. Correlation analysis found a positive correlation between chitinases and *HWP*1, suggesting eDNA may release during the hyphal growth. Finally, we showed a combinational treatment of biofilms with DNase or chitinase inhibitor (acetazolamide) plus amphotericin B significantly improved antifungal susceptibility by 2 to 8 fold.

**Conclusions:**

Collectively, these data show that eDNA release by *C. albicans* clinical isolates is variable and is associated with differential biofilm formation. Digestion of biofilm eDNA by DNase may provide a novel therapeutic strategies to destabilise biofilm growth and improves antifungal sensitivity.

**Electronic supplementary material:**

The online version of this article (doi:10.1186/s12866-014-0303-6) contains supplementary material, which is available to authorized users.

## Background

*Candida albicans* is a dimorphic fungal pathogen that causes both superficial and systemic candidiasis [1]. Invasive systemic forms of the disease affect at-risk patients and can lead to mortality rates as high as 63% in some instances [2]. The disease is multifactorial [3], yet the capacity of *C. albicans* to form biofilms has been identified as a pivotal factor to patient outcomes [4,5]. These structures are found frequently associated with indwelling medical devices, as well as biological surfaces such as the mucosa [6], and are clinically important due to their high recalcitrance to antifungal treatment [7]. However, there is increasing evidence in the literature that *C. albicans* biofilm formation is heterogeneous, which has a direct impact upon treatment and pathogenicity [4,8-10]. Studies have indicated that depending on the ability to form a biofilm, or not, has a bearing on patient outcomes, though the conclusions from some of these studies are diametrically opposed [4,9]. Therefore, rather than defining biofilm formation as an absolute parameter we have recently described a series of clinical isolates with a differential ability to form biofilms, in which high biofilm forming (HBF) isolates displayed a greater pathogenic capacity and lower antifungal sensitivity when compared to low biofilm forming (LBF) isolates [10]. However, the mechanisms underlying the difference in biofilm formation are not yet fully understood.

Extracellular matrix (ECM) is an important and defining characteristic of biofilms, providing a structural scaffold whilst coincidentally facilitating protection from external factors, including antifungal agents [7,11]. Typical fungal biofilm ECM is a heterogeneous substance consisting of exopolysaccharides, proteins, surfactants, lipids and water [12,13], though recent studies have shown the presence of another important component extracellular DNA in fungal biofilm matrix [13,14]. These studies demonstrated that eDNA plays a significant role with respect to structural stability and as a consequence antifungal sensitivity. In both *C. albicans* and *A. fumigatus* it was shown that addition of exogenous DNA significantly improved biofilm formation, and that depletion of biofilm eDNA through the addition of DNase adversely affects the biomass [13,14]. It can be inferred from bacterial biofilm studies that eDNA has a multifactorial purpose, namely as a nutrient source [15], facilitator of genetic information exchange [16], contributor to biofilm stability and dispersal [17-20], and as an antimicrobial resistance factor [21,22]. The mechanism of eDNA release in biofilm environment is not yet fully understood, though studies in bacterial species suggests various mechanisms that are potentially responsible for this process, including cell lysis [23,24]. We recently demonstrated the association of the chitinase regulated autolytic pathway with eDNA release in *A. fumigatus* [14], however, chitinase activity in *C. albicans* biofilms with respect to eDNA has yet to be studied. Given that eDNA contributes to ECM integrity, and ECM is a key factor in promoting biofilm formation, we hypothesised that the capacity to release eDNA through differential chitinase activity may be an underlying mechanism supporting biofilm heterogeneity.

## Methods

### Isolates and maintenance

*C. albicans* SC5314 and a series of routine patient anonymised clinical bloodstream isolates (n = 6) were collected under the approval of the NHS Scotland Caldicott Gaurdians from the Royal Hospital for Sick Children (Yorkhill Division), Glasgow, UK, as part of candidaemia epidemiology surveillance study were used throughout this study. Furthermore, chitinase mutants Δ*cht2,* Δ*cht3,* Δ*cht2/*Δ*cht3*, including the parental strain CA14 were also used. Isolates were stored on Sabouraud dextrose agar (Oxoid, Basingstoke, UK) at 4°C. All *C. albicans* strains were grown on YPD at 37°C overnight. Cells were then washed and resuspended in appropriate media to the desired concentration, as described previously [25]. All procedures were carried out in a laminar flow cabinet (Hera Safe laminate flow cabinet, Kendro, model K515).

### Assessment of biofilm formation

The growth rate of the clinical isolates was first assessed. Each isolate was standardised to 1 × 10^4^ cells/mL in YPD dispensed into each well of a 96 well round-bottom plate and incubated at 37°C for 24 h. The absorbance was measured at 530 nm every 1 h in a microtitre plate reader (FluoStar Omega, BMG Labtech). Each isolate was tested in duplicate and repeated on three independent occasions. Negative controls containing no *C. albicans* were included for background correction. For biofilm screening isolates were processed as described previously [10]. Biofilms were then grown in a 96 well flat-bottomed polystyrene plate (Corning Incorporated, NY, USA) at 37°C for 24 h as described previously [25]. Following incubation, biofilms were carefully washed twice with PBS to remove any non-adherent cells and biomass of each isolate assessed using a crystal violet assay (CV) [26]. Air dried biofilms were stained with 0.05% w/v CV for 20 min, washed to remove excess stain, then destained with 100% ethanol. This was transferred to a new 96 well plate and absorbance was read at 570 nm. Isolates with CV absorbance of ≥1.5 were classified as high biofilm former (HBF) and ≤0.5 were classified as low biofilm former (LBF), as previously described [10].

### Effect of DNase treatment on preformed biofilms

The role of eDNA in *C. albicans* biofilm formation was first investigated by depletion of eDNA within the biofilm using a hydrolytic enzyme DNase I. DNase I from bovine pancreas (Sigma-Aldrich) was prepared in 0.15 M NaCl supplemented with 5 mM of MgCl_2_. Biofilms of HBF (n = 3) and LBF (n = 3) isolates were grown in RPMI-1640 as described above. Following incubation, biofilms were washed with PBS and treated with 256 μg/mL of DNase (Sigma-Aldrich) for 24 h at 37°C, a concentration previously shown to decrease fungal biofilm biomass [14,27]. Untreated controls were also included for direct comparison. Biofilms were then washed twice with PBS and the biomass was scraped from the surface and passed through 0.22 μM membrane filters. Biofilms retained on the filters were dried overnight at 60°C and then dry weight measurements were taken for each isolate, as described previously [28]. Measurement of each isolate was carried out in duplicate, on three independent occasions.

### Assessment of extracellular DNA release

*C. albicans* isolates with LBF (n = 3), HBF (n = 3), CA14, and chitinase mutants Δ*cht2,* Δ*cht3* and Δ*cht2/*Δ*cht3* biofilms were grown in RPMI-1640 for 4 and 24 h at 37°C. The quantity of eDNA release was measured using a microplate fluorescence assay (MFA) using a DNA binding dye (SYBR® Green I), as previously described [14]. Briefly, SYBR® Green I (Invitrogen) was added to biofilm supernatants in a black well microtitre plate (Costar3603; Corning) at a ratio of 1:4. Binding of this dye produces fluorescence directly proportional to DNA concentration. The levels of eDNA were quantified using a fluorescence plate reader (Fluostar Optima; BMG Labtech) at 485 and 518 nm, respectively. The concentration of eDNA in the sample was quantified using the DNA standard curve as previously described [29]. In addition, optical density of the culture was measured at 530 nm simultaneously for normalising the relative fluorescence units (RFU) data. Each isolate was tested in duplicate, on three separate occasions.

### Fluorescence microscopy

Standardised *C. albicans* (1 × 10^6^ cells/ml) were inoculated in RPMI medium onto Thermanox™ coverslips (13 mm) within a 24-well tissue culture plate, then incubated for 24 h at 37°C. These were gently rinsed with PBS and stained according to the manufacturers’ instructions with 5 μM calcofluor white (Invitrogen), which binds chitin and beta-glucans of fungal cell walls, and with 20 μM propidium iodide (PI) (Sigma), which stains the DNA present within a biofilm. Biofilm growth and accumulation of eDNA were visualized under a fluorescence microscope (Motic BA400 Colorview system) at Ex_350_/Em_400_ for calcofluor white, and Ex_540_/Em_525_ for propidium iodide. Representative images from 10 fields were taken and one from each group was digitally photographed.

### Quantifying chitinase activity

*C. albicans* chitinase activity was quantified from isolates with LBF (n = 3) and HBF (n = 3) after 4 and 24 h of biofilm formation using a fluorometric chitinase assay kit (Sigma, United Kingdom), as per the manufacturer’s instructions. Following biofilm development, supernatants were collected at 4 and 24 h, and an appropriate volume of each sample was incubated with a substrate working solution (4-methylumbelliferyl N-acetyl-β-d-glucosaminide) at 37°C for 30 min. Fluorescence was then quantified at Ex_360_/Em_450_. Appropriate positive and negative controls included in the kit were added to each plate. Chitinase activity was calculated and expressed as a ratio between chitinase units and optical density of culture (U/OD). Each isolate was measured in duplicate, on three separate occasions.

### Quantitative gene expression

*C. albicans* HBF (n = 3) and LBF (n = 3) biofilms were prepared in 24 well polystyrene plates as described above for 4 and 24 h at 37°C. Quantitative analysis of transcriptional changes within these biofilms was performed as previously described [30]. Briefly, RNA was extracted by mechanical disruption in TRIzol (Invitrogen) and purified using an RNeasy MinElute cleanup kit (Qiagen, Crawley, United Kingdom) as per the manufacturer’s instructions. RNA was quantified and its quality determined using a NanoDrop spectrophotometer (ND-1000; Thermo Scientific, Loughborough, United Kingdom). Then, cDNA was synthesised with a high-capacity RNA-to-cDNA master mix (Applied Biosystems), using a MyCycler PCR machine (Bio-Rad, Hertfordshire, United Kingdom), and was stored at −20°C for expression analysis. The expression of the chitinase genes *CHT2* and *CHT3* and markers of hyphal cell growth and adhesion (*HWP*1 and *ALS*3*)* were then assessed using quantitative reverse transcription-PCR (RT-PCR) with SYBR® GreenER™ (Invitrogen), according to the manufacturer’s instructions. Primer sequences for these genes are shown in Table 1. The individual gene expression levels were then calculated using the 2^−ΔCT^ method for different phases and normalized to the *ACT1* housekeeping gene. All isolates were tested in duplicate for this experiment.

**Table 1:** ***C. albicans*** **primers used for qPCR**

**Gene**	**Sequence (5′ - 3′)**	**Function**
***ALS3***	F - CAACTTGGGTTATTGAAACAAAAACA	Adhesin
	R - AGAAACAGAAACCCAAGAACAACCT	
***HWP1***	F - GCTCAACTTATTGCTATCGCTTATTACA	Hyphal wall protein
	R - GACCGTCTACCTGTGGGACAGT	
***CHT2***	F – TGATTTATTATCCAAAGTCCCACTTG	Chitinase
	R – TTGAATTGGCCATTGATTGAA	
***CHT3***	F - TGCTACTATTCCAGATGACAAAGAAATT	Chitinase
	R - TTCAGTGATGATAGCAGGTGGTTT	
***ACT1***	F - AAGAATTGATTTGGCTGGTAGAGA	Actin - housekeeping
	R - TGGCAGAAGATTGAGAAGAAGTTT	

### Scanning electron microscopy

*C. albicans* single (Δ*cht2,* Δ*cht3),* double knockout (Δ*cht2/*Δ*cht3*) chitinase mutants and their parental strain CA14 were standardised and grown as biofilm in RPMI-1640 directly onto Thermanox™ coverslips (Nunc, Roskilde, Denmark) for 24 h. Following biofilm development, biofilms were carefully washed with PBS before fixation in 2% para-formaldehyde, 2% gluteraldehyde and 0.15 M sodium cacodylate, and 0.15% w/v Alcian Blue, pH 7.4, and prepared for scanning electron microscopy (SEM), as previously described [31]. The samples were sputter-coated with gold and viewed under a JEOL JSM-6400 SEM. Images were assembled using Photoshop software (Adobe, San Jose, CA, USA) at magnification × 1000.

### Biofilm susceptibility testing in the presence of DNase and azetazolamide

The impact of eDNA and chitinases on amphotericin B sensitivity was assessed using a hydrolytic enzyme DNase I and a chitinase inhibitor acetazolamide ([AZE] Sigma-Aldrich). *C. albicans* LBF (n = 3), HBF (n = 3) and SC5314 were standardized to 1 × 10^6^ cells/ml in RPMI-1640 and biofilms grown in flat bottomed 96 well plates for 4 and 24 h. Preformed biofilms were washed twice with PBS and treated with serially diluted AMB ± DNase (256 μg/ml) or ± AZE (256 μg/ml). The concentrations of DNase and AZE were previously shown to be effectively disrupting the biomass and improve antifungal sensitivity [14,27]. Biofilms were incubated for a further 24 h at 37°C before metabolic activity assessed using the XTT assay, as described previously [32].

### Statistics

Analysis of variance (ANOVA) and t-tests were used to investigate independent sample data. A Bonferroni correction for multiple comparisons was applied to the data where appropriate. SPSS (Version 11, Chicago, USA) was used for these analysis and GraphPad Prism (Version 4, La Jolla, USA) for the production of the figures. The Spearman’s rho correlation coefficient was determined to investigate the relationship between the parameters measured. A *p* value of less than 0.05 was considered significant.

## Results

### *Candida albicans* eDNA release is correlated with biofilm formation and promotes antifungal resistance

**Figure 1:**
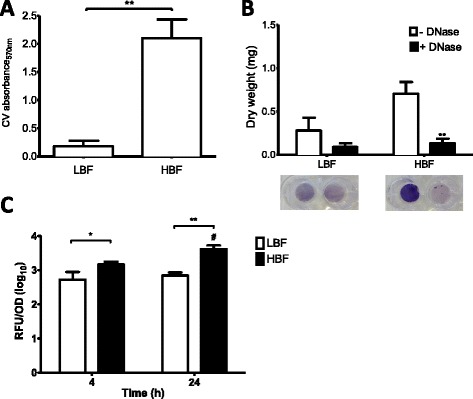
**Variation in** ***C. albicans*** **biofilm formation and eDNA release.** *C. albicans* isolates with LBF (n = 3) and HBF (n = 3) were grown as biofilms in 96 well flat-bottom microtitre plates at 37°C for 24 h. **(A)** Biofilm biomass was assessed spectrophotometrically by reading CV absorbance, data represents mean ± SD. **(B)** Biofilms were treated ± 256 μg/ml DNase for a further 24 h before being passed through 0.22 μM membrane filter. Biomass retained on the filters was dried overnight at 60°C and dry weight measurements taken. In addition, biofilms were stained with CV and imaged to show the disruptive effect of DNase on the biofilms, data represents mean ± SE. **(C)** Isolates were grown as biofilms for 4 and 24 h in the presence of the DNA binding dye SYBR® Green I. Fluorescence was measured after 4 and 24 h at Ex485/Em518. Absorbance was measured simultaneously for normalising the fluorescence data, data represents mean ± SD. Each isolate was tested in duplicate, on three independent occasions. *^#^p < 0.05, **p < 0.01.

Initially, we assessed *C. albicans* biofilm formation by six bloodstream isolates defined as either low biofilm former (LBF, n = 3) or high biofilm former (HBF, n = 3) based on biomass quantification. A significant difference in biofilm formation was observed between isolates with LBF and HBF, where 11.6× more (p = 0.0035) biomass was observed in the latter group (Figure 1A). We then evaluated whether the presence of eDNA contributed to *C. albicans* biofilms formation. To do so we investigated how each LBF and HBF isolate responded to DNase treatment. A significant biomass (dry weight) reduction was observed for the HBF (p < 0.01), with a 5-fold decrease in biomass compared to untreated controls (Figure 1B). In comparison, isolates with the LBF phenotype did not show any significant biomass reduction. To further investigate the hypothesis that eDNA preferentially supported biofilm growth, we evaluated its release from both HBF and LBF at 4 and 24 h using SYBR green I assay. eDNA release was significantly greater in isolates with HBF compared to LBF at both 4 h and (2.78 fold, p < 0.05) and 24 h (5.89 fold, p < 0.01) (Figure 1C). Moreover, eDNA release increased significantly by a further 2.79 fold (p = 0.0221) in HBF isolates between 4 and 24 h. No differences in growth rates were observed between the 6 test isolates (Additional file 1: Figure S1).

Next, the impact of eDNA on AMB sensitivity was then tested to determine its role in antifungal resistance. It was shown that isolates with LBF were up to 8-fold more susceptible to AMB than those with HBF at 4 h. However, when comparing these biofilm subsets at 24 h there was only a 2-fold difference in MIC (Table 2). The addition of DNase during AMB treatment did not have an impact on isolates with LBF at 4 h, with only a 2-fold difference observed at 24 h with DNase treatment. In contrast, isolates with HBF were most susceptible to AMB + DNase therapy at both time points, particularly 24 h where up to 8-fold decrease in MIC were observed in the presence of DNase. The addition of DNase with AMB increased AMB sensitivity by up to 2 and 8-fold in HBF isolates from 4 and 24 h biofilms, respectively, whereas, those LBF isolates showed no change at 4 h, and only a 2 fold change at 24 h (Table 2).

**Table 2:** **MIC of amphotericin B against** ***C. albicans*** **SC5314, LBF and HBF**

**Minimum inhibitory concentrations (MICs)**						
**Strain**	**4 h biofilm**			**24 h biofilm**		
	**AMB MIC**_50_^*^ **(mg/L)**	FC** + DNase	**FC + AZE**	**AMB MIC**_**50**_ **(mg/L)**	**FC + DNase**	**FC + AZE**
**SC5314**	<0.0313	1	1	0.5	4	2
**LBF**	0.0313	1	1	0.25 – 0.5	1 - 2	1
**HBF**	0.125 – 0.25	1 - 2	1	0.25 - 1	4 - 8	2

*MIC_50_ – Minimum concentration of AMB at which 50% reduction in viability assessed by XTT assay.

**FC – Fold change in MIC of AMB in the presence and absence of DNase or acetazolamide (AZE).

### Differential expression of *Candida albicans* chitinase activity is correlated with eDNA release and biofilm formation

**Figure 2:**
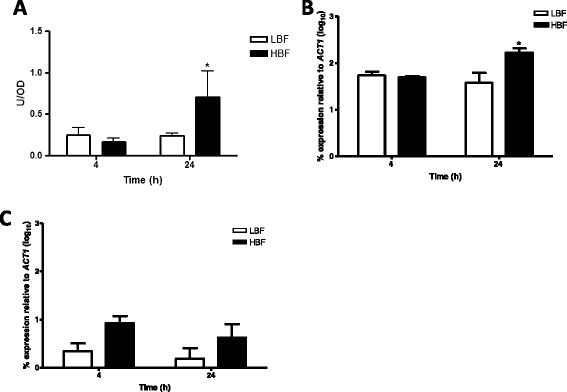
**Chitinases play a role in** ***C. albicans*** **biofilm formation.** Three *C. albicans* isolates with LBF and HBF were standardised to 1 × 10^6^ cells/ml in RPMI-1640 and grown in 24 well microtitre plates at 37°C for 4 and 24 h. **(A)** Supernatants were retained and mixed with a chitinase substrate working solution for 30 min at 37°C. Fluorescence was measured at 360 and 450 nm and chitinase activity represented at U/OD, normalised to isolate biomass. Each isolate was measured in duplicate, on three separate occasions. **(B-C)** Biofilms with LBF (n = 3) and HBF (n = 3) were washed with PBS and RNA extracted using the TRIzol method, cDNA synthesised and real-time PCR used to measure the expression of *CHT2* **(B)** and *CHT3* **(C)**. Percentage of gene expression is shown as log_10_ mean ± SD relative to housekeeping gene *ACT1*. *p < 0.05.

**Figure 3:**
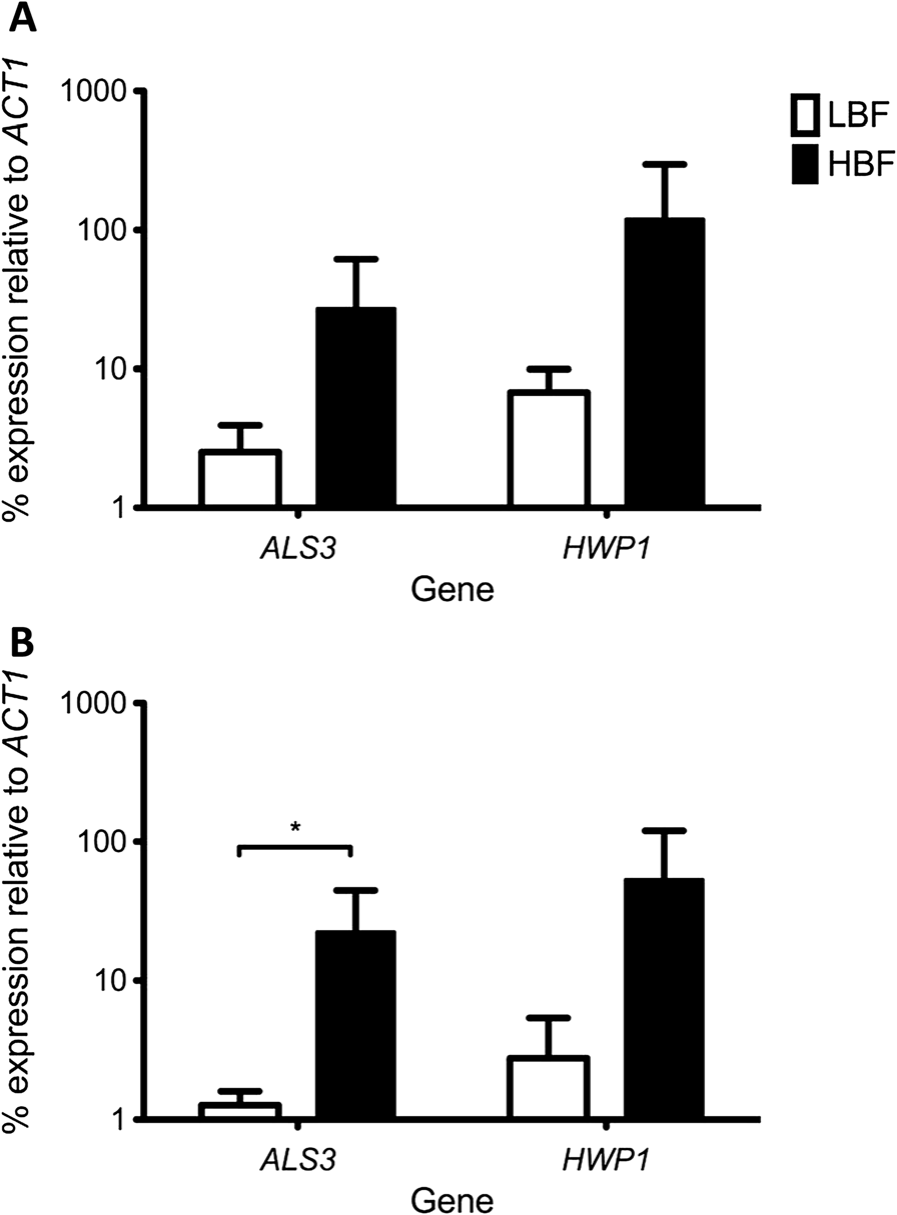
**Expression of biofilm related genes is up-regulated in** ***C. albicans*** **isolates with HBF.** Biofilms with LBF (n = 3) and HBF (n = 3) were grown for 4 **(A)** and 24 h **(B)** before RNA extracted, cDNA synthesised and qPCR used to measure the expression of *ALS3* and *HWP1*. Each individual isolate was measured in duplicate. Percentage of gene expression is represented by mean ± SD relative to housekeeping gene *ACT1*. *p < 0.05.

Based on previous work from our group, which showed a positive correlation between chitinase activity in *Aspergillus fumigatus* biofilms and eDNA release [14], we decided to evaluate whether this property was also observed in *C. albicans* biofilms. Although no differences in chitinase activity were identified with biochemical assay between LBF and HBF isolates at 4 h, a significant change of ~3-fold greater activity detected at 24 h (p < 0.05) with HBF compared to LBF (Figure 2A). Furthermore, transcriptional analysis found no difference in *CHT2* expression at 4 h between the two biofilm subsets, a significant up-regulation by 23% (p < 0.05) was observed at 24 h in those with HBF compared to those with LBF (Figure 2B). In addition, despite *CHT3* expression being increased by 4-fold and 3-fold in isolates with HBF than LBF at 4 and 24 h, respectively, no significant difference between the groups was observed (Figure 2C). In addition, the expression of biofilm-related genes *HWP1* and *ALS3* in *C. albicans* biofilms was evaluated and it was found that these were up-regulated in isolates with HBF. Despite *ALS3* and *HWP1* expression being increased in HBF by 11× (p = 0.1143) and 18× (p = 0.1447) at 4 h (Figure 3A) when compared to LBF respectively, the only significant difference in the transcriptional analysis was observed at 24 h where LBF had 18× less expression of *ALS3* (p = 0.0102) than HBF (Figure 3B).

To investigate the relationship between eDNA release, chitinase activity, biofilm biomass and genes related to biofilm formation (*HWP1* and *ALS*3), a Spearman’s rho correlation analysis was performed (Table 3). A significant correlation was observed between eDNA release and biomass (p < 0.01), chitinase activity (p < 0.01), and expression of *HWP1* (p < 0.05) and *ALS3* (p < 0.05) at 24 h. A significant positive correlation was also found between chitinase activity and expression of *HWP1* (p < 0.05) in 24 h biofilms (Table 3).

**Table 3:** **Spearman’s rho correlation analysis**

**Spearman’s rho**		**Biomass**	**Chitinase**	***HWP1***	***ALS3***
**eDNA**	Correlation Coefficient	.829^*^	.771^*^	.886^**^	.829^*^
	Sig.	.021	.036	.009	.021
**Chitinase**	Correlation Coefficient	.771^*^		.771^*^	.600
	Sig.	.036		.036	.104

*Correlation is significant at the 0.05 level.

**Correlation is significant at the 0.01 level.

### Compromising *Candida albicans* chitinase activity affects isolates biofilm formation and antifungal sensitivity

**Figure 4:**
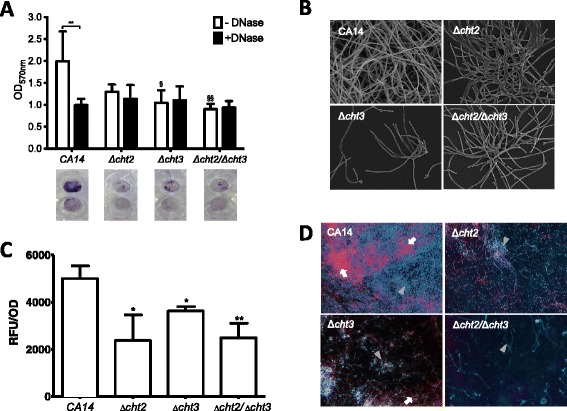
**Compromising chitinase activity decreases eDNA release and biofilm formation in** ***C. albicans*** **.** *C. albicans* Δ*cht2*, Δ*cht3*, Δ*cht2/*Δ*cht3* and parental strain CA14 biofilms were grown in 96 well microtitre plates for 24 h at 37°C in RPMI **(A)** Following biofilm development, biofilms were carefully washed with PBS and incubated in RPMI for a further 24 h, ± 256 μg/ml DNase. Biomass assessed spectrophotometrically by measuring CV absorbance at 570 nm. Also CV stained biofilms were digitally imaged to show the difference in biofilm formation and effect of DNase treatment. §*P < 0.05, §§ p < 0.01*. **(B)** *C. albicans* Δ*cht2,* Δ*cht3,* Δ*cht2/*Δ*cht3* and wild type CA14 (×1000) were grown on Thermanox™ coverslips for 24 h at 37°C. Biofilms were then processed and viewed on a JEOL JSM-6400 scanning electron microscope and images assembled using Photoshop software. Note the reduction in biofilm biomass in Δ*cht3* and Δ*cht2/*Δ*cht3*. **(C)** eDNA release was measured after 4 and 24 h using SYBR green I assay, normalised to each isolates biomass. Each isolate was measured in duplicate, on three separate occasions. *p < 0.05, **p < 0.01. **(D)** Biofilm growth (hyphal cells indicated by arrow head) and accumulation of eDNA (Indicated by arrow) were visualized under a fluorescence microscope (Motic BA400 Colorview system) at an Ex/Em wavelengths of 350/400 nm for calcofluor white and 540/525 nm for propidium iodide. One representative from each group was digitally photographed.

*C. albicans* single and double knockout mutants Δ*cht2,* Δ*cht3* and Δ*cht2/*Δ*cht3* were assessed to confirm whether these chitinases played a role in eDNA release and biofilm formation. These all showed a reduction in biofilm biomass when compared to their parental strain CA14. However, a significant reduction was found only with Δ*cht3* (p < 0.05) and Δ*cht2/*Δ*cht3* (p < 0.005) (Figure 4A). To investigate whether this reduction in biomass is caused by presence of eDNA, the biofilms were treated with DNase. Here a significant reduction in biofilm biomass by 2-fold was observed with DNase treatment in CA14 compared to untreated controls (p < 0.01). Conversely, no significant reduction was observed with either single or double knockout mutants (Figure 4A). The reduction in biofilm formation by all the knockout mutants was confirmed by microscopy (Figure 4B). SEM imaging showed reduced biomass in these isolates compared to CA14, with Δ*cht3* showing the clearest visual difference, providing evidence that chitinases plays a role in *C. albicans* biofilm formation. eDNA release was also quantified in these mutants. All knockout strains had significantly reduced eDNA release after 24 h. The greatest reduction was in Δ*cht2*, where a significant eDNA reduction of 53% (p < 0.05) was measured compared to the wild-type, followed by Δ*cht2/*Δ*cht3* with reduction of 50% (p = 0.0061), and Δ*cht3* where eDNA release was reduced by 26% (p = 0.0161) (Figure 4C). The level of eDNA present in these knockout mutants was confirmed visually using fluorescence imaging. The presence of eDNA in the parental strain CA14 is shown by the abundance of red/pink fluorescence, which was notably reduced in the single knockouts Δ*cht2* and Δ*cht3*, and completely absent from the double knockout Δ*cht2*/Δ*cht3* (Figure 4D).

Finally, to test whether pharmacological inhibition of chitinases impacted antifungal sensitivity, AZE was used in combination with AMB. It was shown that the addition of AZE did not alter AMB sensitivity in any of the LBF biofilms at 4 h and 24 h. However, isolates with HBF had up to a 2 fold decrease in MIC_50_ at both 4 and 24 h (Table 2).

## Discussion

Our group have recently described that biofilms formed by different clinical isolates of *C. albicans* are heterogeneous. Significant variation in their ability to form and maintain biofilm structure exists between bloodstream isolates, which was shown not only to impact biofilm formation, but also pathogenicity and antifungal sensitivity [10]. Understanding why these isolates differ would be useful in developing better management strategies, both in terms of antifungal therapy, but potentially also for development of new molecular diagnostics. The data presented herein describes a novel role for eDNA release in conferring heterogeneity during biofilm formation by *C. albicans*, which is associated with chitinase regulated autolytic events, and which contributes to biofilm associated antifungal resistance.

There have been limited studies to date examining the presence and role of eDNA in *C. albicans* biofilms [13,27,33]. These studies demonstrate that the quantity of eDNA in biofilms varied considerably with the growth medium used, with RPMI showing a significantly higher accumulation of eDNA in ECM compared to other tested media [13]. We reported that RPMI consistently supports optimal biofilm formation for *C. albicans* [10]. Given that eDNA was associated with high levels of biofilm formation it was hypothesised that this may explain the underlying mechanism for the heterogeneity, i.e. those isolates with the propensity to form biofilms may release more eDNA than isolates forming structurally simple biofilms. This is supported by the data showing that addition of exogenous DNA has been shown to significantly improve biofilm biomass [14,34]. Moreover, there are studies highlighting the importance of eDNA within mixed *C. albicans* and bacterial biofilms [33,35]. To test these hypotheses two groups of contrasting biofilm forming abilities were investigated. Based on two biomass quantification assays the isolates were categorised as LBF and HBF, from which eDNA release was initially evaluated. Here the levels of eDNA observed were greater in isolates with HBF, which also increased with biofilm maturity, which is a similar finding to that reported in studies of *A. fumigatus* biofilm development [14].

The release of eDNA in fungal biofilms is hypothesised to be an end product of autolysis, a process controlled by various hydrolases, including chitinases [14,36]. *C. albicans* have also been shown to possess complex chitinase families that hydrolyse chitin molecules in fungal cell wall [37]. These enzymes are known to be involved in spore formation, hyphal growth, hyphal branching and septum formation. There are four chitinase genes known in *C. albicans* (*CHT1-4*)*,* though their function remains unclear. *CHT2* and *CHT3* have been shown to be associated with yeast to hyphal morphogenesis and are more active in the hyphal form [38]. Here it was reported that these enzymes were transcriptionally expressed and biochemically active during the release of eDNA, which in turn contributes towards biofilm formation. Indeed, correlation analysis demonstrates the functional relationship between eDNA release, chitinase activity and biofilm formation. Furthermore, a positive correlation between chitinase activity and a hyphal cell marker *HWP1* suggest the involvement of chitinases in hyphal growth, which in turn contributes to eDNA release. This may explain the morphological differences between the HBF and LBF, of which the former demonstrates more filamentous growth [10]. It was also shown that compromising chitinase activity through gene deletion of Δ*cht2,* Δ*cht3* and Δ*cht2/*Δ*cht3* significantly affects eDNA release and biofilm formation, which suggests a role for chitinases in the maintenance and architecture of *C. albicans* biofilms.

From a translational perspective, the mechanism of eDNA release could represent an Achilles heal in the management of tenacious HBF isolates. eDNA release was shown to improve AMB resistance, demonstrated through DNase combinational treatments and also through pharmacological inhibition of chitinases that may regulate eDNA release. Collectively, these data demonstrate the possibility of improving our treatment of these infections through rationally designed strategies. Mismanagement also has important repercussions, as it has been shown that treatment with sub-MIC levels of antifungals causes an increase in chitin content within *Candida* species, which relates to decreased antifungal sensitivity [39,40]. Moreover, a recent study has shown the use of sub-lethal concentrations of antibiotics leads to increased autolysis, eDNA release and biofilm formation in bacteria [41]. Tumbarello and colleagues identified inadequate antifungal therapy as a predictor of patient mortality [42], with other studies highlighting the importance of efficient and appropriate treatment of candidaemia cases [43,44]. Therefore, careful consideration must be given with regards to antifungal use as ineffectual therapy can lead to increased antifungal resistance and increased hospital lengths of stay.

## Conclusions

The data from this study showed a variation in biofilm formation among *C. albicans* isolates and how it affects the antifungal sensitivity. Strain dependent eDNA release and chitinase activity is associated with biofilm formation. Finally, pharmacological inhibition of eDNA by DNase or AZE reduced biofilm resistance to AMB in *C. albicans*. Thus, we establish a potential mechanism regulating biofilm heterogeneity and antifungal resistance, and that targeting eDNA may provide an effective strategy for management of biofilm based infections.

## Additional file

Additional file 1: Figure S1 Variation in *Candida albicans* biofilm formation is independent of growth kinetics. The growth kinetics of *C. albicans* isolates with LBF (grey circle) and HBF (black square) was assessed over 24 h, with absorbance read at 530 nm every hour. Isolates with LBF (n = 3) and HBF (n = 3) were grown in duplicate, on three separate occasions. Data represents mean value.

## Abbreviations

ACT: Actin
CHT: Chitinase
ALS: Aglutanin like sequence
AMB: Amphotericin B
ANOVA: Analysis of variance
AZE: Acetazolamide
CV: Crystal violet
ECM: Extracellular matrix
eDNA: Extracellular DNA
Em: Emission
Ex: Excitation
HBF: High biofilm former
HWP: Hyphal wall protein
LBF: Low biofilm former
MIC: Minimum inhibitory concentration
PBS: Phosphate buffered saline
qPCR: Quantitative polymerase chain reaction
RFU: Relative fluorescence units
RPMI: Roswell park memorial institute
SD: Standard deviation
SE: Standard error of the mean
SEM: Scanning electron microscopy
U/OD: Chitinase units per optical density
XTT: 2,3-bis(2-methoxy-4-nitro-5-sulfo-phenyl)-2H-tetrazolium-5-caboxanilide
YPD: Yeast peptone dextrose

## References

[1] Odds FC (1987). Candida infections: an overview. Crit Rev Microbiol.

[2] Kollef M, Micek S, Hampton N, Doherty JA, Kumar A (2012). Septic shock attributed to Candida infection: importance of empiric therapy and source control. Clin Infect Dis.

[3] Eggimann P, Bille J, Marchetti O: **Diagnosis of invasive candidiasis in the ICU.** *Ann Intensive Care* 2011, **1:**37. 10.1186/2110-5820-1-37 PMC3224461 21906271

[4] Tumbarello M, Fiori B, Trecarichi EM, Posteraro P, Losito AR, De Luca A, Sanguinetti M, Fadda G, Cauda R, Posteraro B: **Risk factors and outcomes of candidemia caused by biofilm-forming isolates in a tertiary care hospital.** *PLoS One* 2012, **7**(3):e33705. 10.1371/journal.pone.0033705 PMC3316499 22479431

[5] Andes DR, Safdar N, Baddley JW, Playford G, Reboli AC, Rex JH, Sobel JD, Pappas PG, Kullberg BJ (2012). Impact of treatment strategy on outcomes in patients with candidemia and other forms of invasive candidiasis: a patient-level quantitative review of randomized trials. Clin Infect Dis.

[6] Ramage G, Williams C (2013). The clinical importance of fungal biofilms. Adv Appl Microbiol.

[7] Ramage G, Rajendran R, Sherry L, Williams C: **Fungal biofilm resistance.** *Int J Microbiol* 2012, **2012:**528521. 10.1155/2012/528521 PMC3299327 22518145

[8] Alnuaimi AD, O’Brien-Simpson NM, Reynolds EC, McCullough MJ (2013). Clinical isolates and laboratory reference Candida species and strains have varying abilities to form biofilms. FEMS Yeast Res.

[9] Guembe M, Guinea J, Marcos-Zambrano L, Fernandez-Cruz A, Pelaez T, Munoz P, Bouza E (2014). Is biofilm production a predictor of catheter-related candidemia?. Med Mycol.

[10] Sherry L, Rajendran R, Lappin DF, Borghi E, Perdoni F, Falleni M, Tosi D, Smith K, Williams C, Jones B, Nile CJ, Ramage G: **Biofilms formed by Candida albicans bloodstream isolates display phenotypic and transcriptional heterogeneity that are associated with resistance and pathogenicity.** *BMC Microbiol* 2014, **14**(1):182. 10.1186/1471-2180-14-182 PMC4105547 24996549

[11] Flemming HC, Wingender J (2010). The biofilm matrix. Nat Rev Microbiol.

[12] Al-Fattani MA, Douglas LJ (2006). Biofilm matrix of Candida albicans and Candida tropicalis: chemical composition and role in drug resistance. J Med Microbiol.

[13] Martins M, Uppuluri P, Thomas DP, Cleary IA, Henriques M, Lopez-Ribot JL, Oliveira R (2010). Presence of extracellular DNA in the Candida albicans biofilm matrix and its contribution to biofilms. Mycopathologia.

[14] Rajendran R, Williams C, Lappin DF, Millington O, Martins M, Ramage G (2013). Extracellular DNA release acts as an antifungal resistance mechanism in mature Aspergillus fumigatus biofilms. Eukaryot Cell.

[15] Mulcahy H, Charron-Mazenod L, Lewenza S (2010). *Pseudomonas aeruginosa* produces an extracellular deoxyribonuclease that is required for utilization of DNA as a nutrient source. Environ Microbiol.

[16] Molin S, Tolker-Nielsen T (2003). Gene transfer occurs with enhanced efficiency in biofilms and induces enhanced stabilisation of the biofilm structure. Curr Opin Biotechnol.

[17] Whitchurch CB, Tolker-Nielsen T, Ragas PC, Mattick JS: **Extracellular DNA required for bacterial biofilm formation.** *Science* 2002, **295**(5559):1487. 10.1126/science.295.5559.1487 11859186

[18] Izano EA, Amarante MA, Kher WB, Kaplan JB (2008). Differential roles of poly-N-acetylglucosamine surface polysaccharide and extracellular DNA in *Staphylococcus aureus* and *Staphylococcus epidermidis* biofilms. Appl Environ Microbiol.

[19] Conover MS, Mishra M, Deora R: **Extracellular DNA is essential for maintaining Bordetella biofilm integrity on abiotic surfaces and in the upper respiratory tract of mice.** *PLoS One* 2011, **6**(2):e16861. 10.1371/journal.pone.0016861 PMC3037945 21347299

[20] Berne C, Kysela DT, Brun YV (2010). A bacterial extracellular DNA inhibits settling of motile progeny cells within a biofilm. Mol Microbiol.

[21] Mulcahy H, Charron-Mazenod L, Lewenza S: **Extracellular DNA chelates cations and induces antibiotic resistance in*****Pseudomonas aeruginosa*****biofilms.** *PLoS Pathog* 2008, **4**(11):e1000213. 10.1371/journal.ppat.1000213 PMC2581603 19023416

[22] Tetz GV, Artemenko NK, Tetz VV (2009). Effect of DNase and antibiotics on biofilm characteristics. Antimicrob Agents Chemother.

[23] Allesen-Holm M, Barken KB, Yang L, Klausen M, Webb JS, Kjelleberg S, Molin S, Givskov M, Tolker-Nielsen T (2006). A characterization of DNA release in Pseudomonas aeruginosa cultures and biofilms. Mol Microbiol.

[24] Qin Z, Ou Y, Yang L, Zhu Y, Tolker-Nielsen T, Molin S, Qu D (2007). Role of autolysin-mediated DNA release in biofilm formation of Staphylococcus epidermidis. Microbiology.

[25] Ramage G, Vande Walle K, Wickes BL, Lopez-Ribot JL (2001). Standardized method for in vitro antifungal susceptibility testing of Candida albicans biofilms. Antimicrob Agents Chemother.

[26] Mowat E, Butcher J, Lang S, Williams C, Ramage G (2007). Development of a simple model for studying the effects of antifungal agents on multicellular communities of Aspergillus fumigatus. J Med Microbiol.

[27] Martins M, Henriques M, Lopez-Ribot JL, Oliveira R (2012). Addition of DNase improves the in vitro activity of antifungal drugs against Candida albicans biofilms. Mycoses.

[28] Richard ML, Nobile CJ, Bruno VM, Mitchell AP (2005). Candida albicans biofilm-defective mutants. Eukaryot Cell.

[29] Leggate J, Allain R, Isaac L, Blais BW (2006). Microplate fluorescence assay for the quantification of double stranded DNA using SYBR Green I dye. Biotechnol Lett.

[30] Ramage G, Coco B, Sherry L, Bagg J, Lappin DF (2012). In vitro Candida albicans biofilm induced proteinase activity and SAP8 expression correlates with in vivo denture stomatitis severity. Mycopathologia.

[31] Erlandsen SL, Kristich CJ, Dunny GM, Wells CL (2004). High-resolution visualization of the microbial glycocalyx with low-voltage scanning electron microscopy: dependence on cationic dyes. J Histochem Cytochem.

[32] Pierce CG, Uppuluri P, Tristan AR, Wormley FL, Mowat E, Ramage G, Lopez-Ribot JL (2008). A simple and reproducible 96-well plate-based method for the formation of fungal biofilms and its application to antifungal susceptibility testing. Nat Protoc.

[33] Sapaar B, Nur A, Hirota K, Yumoto H, Murakami K, Amoh T, Matsuo T, Ichikawa T, Miyake Y (2014). Effects of extracellular DNA from Candida albicans and pneumonia-related pathogens on Candida biofilm formation and hyphal transformation. J Appl Microbiol.

[34] Shopova I, Bruns S, Thywissen A, Kniemeyer O, Brakhage AA, Hillmann F: **Extrinsic extracellular DNA leads to biofilm formation and colocalizes with matrix polysaccharides in the human pathogenic fungus Aspergillus fumigatus.** *Front Microbiol* 2013, **4:**141. 10.3389/fmicb.2013.00141 PMC3674311 23760756

[35] Pammi M, Liang R, Hicks J, Mistretta TA, Versalovic J: **Biofilm extracellular DNA enhances mixed species biofilms of Staphylococcus epidermidis and Candida albicans.** *BMC Microbiol* 2013, **13:**257. 10.1186/1471-2180-13-257 PMC3833181 24228850

[36] White S, McIntyre M, Berry DR, McNeil B (2002). The autolysis of industrial filamentous fungi. Crit Rev Biotechnol.

[37] Gooday GW, Wei-Yun Z, O’Donnell RW (1992). What are the roles of chitinases in the growing fungus?. FEMS Microbiol Lett.

[38] Selvaggini S, Munro CA, Paschoud S, Sanglard D, Gow NA (2004). Independent regulation of chitin synthase and chitinase activity in Candida albicans and Saccharomyces cerevisiae. Microbiology.

[39] Walker LA, Gow NA, Munro CA (2013). Elevated chitin content reduces the susceptibility of Candida species to caspofungin. Antimicrob Agents Chemother.

[40] Walker LA, Munro CA, de Bruijn I, Lenardon MD, McKinnon A, Gow NA: **Stimulation of chitin synthesis rescues Candida albicans from echinocandins.** *PLoS Pathog* 2008, **4**(4):e1000040. 10.1371/journal.ppat.1000040 PMC2271054 18389063

[41] Hsu CY, Lin MH, Chen CC, Chien SC, Cheng YH, Su IN, Shu JC (2011). Vancomycin promotes the bacterial autolysis, release of extracellular DNA, and biofilm formation in vancomycin-non-susceptible Staphylococcus aureus. FEMS Immunol Med Microbiol.

[42] Tumbarello M, Posteraro B, Trecarichi EM, Fiori B, Rossi M, Porta R, de Gaetano DK, La Sorda M, Spanu T, Fadda G, Cauda R, Sanguinetti M (2007). Biofilm production by Candida species and inadequate antifungal therapy as predictors of mortality for patients with candidemia. J Clin Microbiol.

[43] Almirante B, Rodriguez D, Park BJ, Cuenca-Estrella M, Planes AM, Almela M, Mensa J, Sanchez F, Ayats J, Gimenez M, Saballs P, Fridkin SK, Morgan J, Rodriguez-Tudela JL, Warnock DW, Pahissa A (2005). Epidemiology and predictors of mortality in cases of Candida bloodstream infection: results from population-based surveillance, barcelona, Spain, from 2002 to 2003. J Clin Microbiol.

[44] Garey KW, Rege M, Pai MP, Mingo DE, Suda KJ, Turpin RS, Bearden DT (2006). Time to initiation of fluconazole therapy impacts mortality in patients with candidemia: a multi-institutional study. Clin Infect Dis.

