# Supplementary figures and images for: Extracellular DNA release confers heterogeneity in Candida albicans biofilm formation
Source: BMC Microbiol. 2014 Dec 5;14:303. doi: 10.1186/s12866-014-0303-6 (PMC4262977; doi:10.1186/s12866-014-0303-6)

## Slide 1
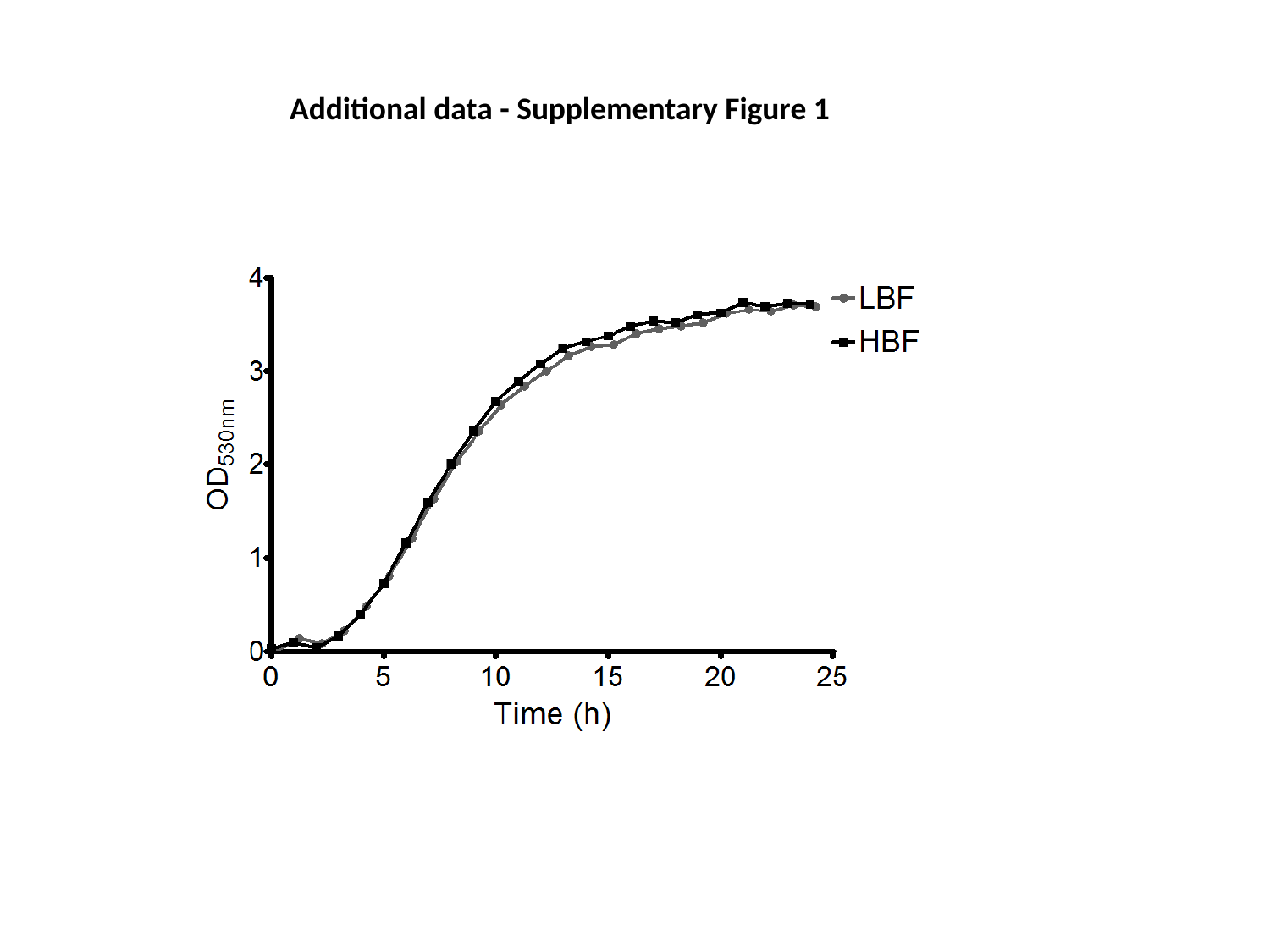

Additional data - Supplementary Figure 1

Supplement: Additional file 1: Figure S1 — Variation in Candida albicans biofilm formation is independent of growth kinetics. The growth kinetics of C. albicans isolates with LBF (grey circle) and HBF (black square) was assessed over 24 h, with absorbance read at 530 nm every hour. Isolates with LBF (n = 3) and HBF (n = 3) were grown in duplicate, on three separate occasions. Data represents mean value. [file 12866_2014_303_MOESM1_ESM.pptx]
